# Predicting novel biomarkers for early diagnosis and dynamic severity monitoring of human ulcerative colitis

**DOI:** 10.3389/fgene.2024.1429482

**Published:** 2024-07-31

**Authors:** Yu Chen, Li Chen, Sheng Huang, Li Yang, Li Wang, Feiyun Yang, Jinxiu Huang, Xiuliang Ding

**Affiliations:** ^1^ Animal Nutrition Institute, Chongqing Academy of Animal Science, Chongqing, China; ^2^ Institute of Nutrition and Feed, National Center of Technology Innovation for Pigs, Chongqing, China

**Keywords:** DSS-induced mouse model, ulcerative colitis, biomarkers, transcriptome sequencing, early diagnosis, dynamic severity monitoring

## Abstract

**Background:**

Ulcerative colitis is an emerging global health concern that poses a significant threat to human health and can progress to colorectal cancer if not diagnosed and treated promptly. Currently, the biomarkers used clinically for diagnosis and dynamic severity monitoring lack disease specificity.

**Methods:**

Mouse models induced with 2%, 2.5%, and 3% DSS were utilized to simulate human UC with varying severities of inflammation. Transcriptome sequencing technology was employed to identify differentially expressed genes (DEGs) between the control group and each treatment group. Functional enrichment analysis of the KEGG database was performed for shared DEGs among the three treatment groups. DEGs that were significantly and strongly correlated with DSS concentrations were identified using Spearman correlation analysis. Human homologous genes of the interested DEGs were searched in the HomoloGene database, and their regulation patterns in UC patients were validated using the GSE224758 dataset. These genes were then submitted to the DisGeNET database to identify their known associations with human diseases. Online tools, including SignalP 6.0 and DeepTMHMM 1.0, were used to predict signal peptides and transmembrane helices in the amino acid sequences of human genes homologous to the DEGs of interest.

**Results:**

A total of 1,230, 995, and 2,214 DEGs were identified in the 2%, 2.5%, and 3% DSS-induced groups, respectively, with 668 DEGs common across all three groups. These shared DEGs were primarily associated with signaling transport, pathogenesis, and immune response. Through extensive screening, *LGI2* and *PRSS22* were identified as potentially novel biomarkers with higher specificity and ease of detection for the early diagnosis and dynamic severity monitoring of human UC, respectively.

**Conclusion:**

We have identified two potentially novel biomarkers, *LGI2* and *PRSS22*, which are easy of detection and more specific for human UC. These findings provide new insights into the accurate diagnosis and dynamic monitoring of this persistent disease.

## 1 Introduction

Ulcerative colitis (UC) is a chronic, non-specific inflammatory disease of the intestines, primarily affecting the mucosa of the colon and rectum (Gros and Kaplan, 2023). Clinically, it presents as abdominal pain, diarrhea, and stools mixed with mucus, pus, and blood (Guo and Wang, 2023). Atypical symptoms such as skin lesions and growth restriction may also occur in children (Yu and Rodriguez, 2017). UC is characterized by its recurrent nature and incurability (Le Berre et al., 2023). Compared to the general population, patients with UC have a 1.7-fold higher risk of developing colorectal cancer (Gros and Kaplan, 2023) and a reduced average life expectancy of approximately 5 years (Kuenzig et al., 2020). Historically prevalent in Western countries (Studd et al., 2016), UC incidence is now rapidly increasing in East Asian countries due to changes in diet and lifestyle (Ng et al., 2016), making it a global health threat (Kaplan and Ng, 2017; Li et al., 2023; Zhou et al., 2023). Early and accurate diagnosis is imperative for controlling the progression of UC.

The clinical diagnosis of UC typically involves a combination of gastrointestinal symptoms, colonoscopy or sigmoidoscopy with biopsies, and biomarkers (Golovics et al., 2022; Singh et al., 2023) such as C-reactive protein (CRP) (Ishida et al., 2021), erythrocyte sedimentation rate (ESR) (Alper et al., 2017) and fecal calprotectin (FC) (Kawashima et al., 2023). However, early-stage UC may not present obvious clinical symptoms. While colonoscopy or sigmoidoscopy is the most accurate method of examining colonic inflammation (Spiceland and Lodhia, 2018), these procedures are invasive and uncomfortable for patients. Although intestinal ultrasound is a new screening tool (de Voogd et al., 2022), it is not universally applicable, particularly for obese UC patients. In contrast, biomarkers are non-invasive, sensitive, disease-specific, easy to perform, and low-cost. CRP and ESR levels are moderately correlated with UC endoscopic activity, with correlation coefficients of 0.5 and 0.4, respectively. Their sensitivity and specificity for UC ranged from 50.5% to 53.3% and 68.7%–71.3% for CRP, and 85.1% to 87.2% and 63.4% to 66.4% for ESR (Yoon et al., 2014). These findings suggest that CRP or ESR alone is insufficient to accurately reflect endoscopic severity. FC, a marker for neutrophilic intestinal inflammation, has a higher correlation with endoscopic activity than CRP and ESR (Turner et al., 2021) but is not specific for UC. Its use has been investigated in other specific disorders (Burri and Beglinger, 2014), including colorectal cancer (Ye et al., 2018). Therefore, there is a need for novel biomarkers with improved sensitivity, specificity, and potential for early diagnosis and assessment of inflammation severity in UC.

Mice are efficient and well-parallelized animal models due to their small size, short generation cycles, and minimal inter-individual differences (Justice and Dhillon, 2016). Their physiology, biochemistry, and developmental processes are similar to those of humans, with 98% of their genomes being homologous to humans. Consequently, many mouse models can realistically simulate the pathogenesis of human diseases (Celhar and Fairhurst, 2017; Sarkar and Heise, 2019) and their responses to drugs (Costa et al., 2018). The dextran sulfate sodium (DSS)-induced mouse model, which closely mimics human UC in terms of intestinal lesions, is widely used for studying pathogenesis and evaluating drug efficacy in human UC (Li et al., 2019; Arda-Pirincci and Aykol-Celik, 2020; Huang et al., 2020; Lin et al., 2020). A previous study observed that the severity of UC inflammation aggravated with increasing concentrations of DSS (Almutary et al., 2023), suggesting that human UC with different inflammation severities can be mimicked by establishing mouse models induced by varying DSS concentrations.

Transcriptome sequencing technology, which provides data on gene expression levels across all genes in a sample, is a powerful tool for identifying changes in gene expression profiles under different conditions, such as health or disease (Yang et al., 2016). It offers comprehensive insights into genes with significant expression changes, likely involved in disease pathogenesis (Mutryn et al., 2015; Jung et al., 2017) and serves as a method for discovering novel biomarkers or therapeutic targets (Satoh et al., 2014; Kohli et al., 2015; Liang et al., 2015). Therefore, it is possible to combine mouse models induced by different DSS concentrations with transcriptome sequencing to explore potentially novel biomarkers suitable for early diagnosis and inflammation severity assessment of human UC.

In the current study, a mouse UC model induced by 2%, 2.5%, and 3% DSS was established as a research platform. Differentially expressed genes (DEGs) were comprehensively revealed using transcriptome sequencing technology and comparative analysis. These DEGs were subsequently filtered based on criteria such as consistency of regulation patterns in UC patients, sensitivity to mild UC, strong correlation with DSS concentrations, minimal association with other human diseases, and secretability. The aim was to uncover novel potential biomarkers with properties specific to human UC and ease of detection, providing new insights into the accurate diagnosis and monitoring of human UC.

## 2 Materials and methods

### 2.1 Animal experimentation and ethic statement

Thirty-two healthy, 8-week-old male C57BL/6J mice, each weighing 20–22 g, were acquired from SiPeiFu Biotechnology Co. Ltd. (Beijing, China). The mice were housed under standard conditions (26°C, ventilated, clean environment) with *ad libitum* access to food and distilled water. After a 1-week acclimatization period, the mice were randomly divided into four groups of eight, ensuring no significant differences in body weight among the groups. The experimental design included a control group receiving distilled water and three treatment groups administered distilled water containing 2%, 2.5%, and 3% DSS (MP Biomedicals, United States), respectively. All groups were maintained under consistent environmental conditions for an additional week. The animal study protocol above was approved by the Ethics Committee of the Chongqing Academy of Animal Science (protocol code SM20210076, approved on 18 March 2021).

### 2.2 Assessing general condition and disease activity index in mice

The body weights of the mice were recorded daily at a consistent time. The weight loss rate was then calculated and scored as S1 according to the criteria outlined in Table 1. Stool characteristics were observed and scored as S2, also based on the criteria in Table 1. The presence of occult blood in feces was evaluated using a detection kit and scored as S3, following the same table. The Disease Activity Index (DAI) was determined using the equation DAI = $\frac{S1+S2+S3}{3}$ .

**TABLE 1:** Scoring criteria for the rate of weight loss, stool traits, and the extent of occult blood.

Score	Weight loss (%)	Stool traits	The extent of occult blood
0	0	Normal	No blue-green coloration within 3 min
1	1∼5	Loose	Gradually blue coloration in 1–2 min
2	6∼10	Semi-sloppy	Blue-green coloration within 1 min
3	11∼15	Watery	Blue-green coloration within 10 s
4	>15	Severe diarrhea	Immediately dark blue coloration

### 2.3 Harvesting and damage evaluating colon tissues

Upon completion of the experimental modeling, the mice were euthanized via cervical dislocation. The colon tissues were then excised, gently rinsed with sterile saline, and dabbed dry with paper towels. Subsequently, the length of the colon tissue was measured and documented. Each colon was sectioned into four equal parts. Approximately 1 cm from the third segment of each colon was fixed in 4% neutral-buffered formalin, followed by paraffin embedding. The sections were stained with hematoxylin and eosin (H&E) for histological examination. Images of these sections were acquired using a Pannoramic 250 digital slide scanner to assess the extent of lesions within the colon tissue. Lesion scoring was conducted based on the criteria outlined in Table 2.

**TABLE 2:** Scoring criteria for colon tissue injury.

Score	Inflammatory cell infiltration degree	Degree of inflammatory infiltration	Degree of intestinal crypt destruction	Range of lesions (%)
0	No	No	No	No
1	Light	Mucous membrane layer	1/3 crypt of basal destroyed	1–25
2	Middle	Sub-mucous membrane layer	2/3 crypt of basal destroyed	26–50
3	Heavy	Muscular and plasma membrane layers	Complete surface epithelium only	51–75
4	—	—	All crypt and surface epithelium destroyed	76–100

### 2.4 RNA extraction and sequencing of colon tissues

Total RNA was extracted from the second segment of colon tissue using TRIzol (Thermo Fisher Scientific). The concentration, purity, and integrity of the RNA were evaluated using a Nanodrop ND-1000 Spectrophotometer (Thermo Scientific, Wilmington, DE, United States) and an Agilent Bioanalyzer 2100 (Agilent, CA, United States). Six total RNA samples were randomly selected from the control group and each of the treatment groups (Supplementary Table S1) and sent to MajorBio Biomedical Technology Co., Ltd. (Shanghai, China) for mRNA library construction according to following steps: 1) Isolation of mRNA using magnetic beads coated with Oligo (dT); 2) Fragmentation of mRNA by adding fragmentation buffer, followed by isolation of approximately 300 bp fragments through magnetic bead screening; 3) Synthesis of double-stranded cDNA using the small mRNA fragments as templates, employing reverse transcriptase and six-base random primers; 4) End repair of double-stranded cDNA, addition of an “A” base at the 3′ end, and ligation to the Y-junction adapter; 5) Enrichment of the library via PCR amplification and isolation of target bands through agarose gel electrophoresis; 6) Quality checking of the library using an Agilent Bioanalyzer 2100 (Agilent, CA, United States). Subsequently, paired-end (PE) sequencing of the mRNA libraries was performed on the Illumina Novaseq 6000 platform.

### 2.5 Transcriptomic data processing and analysis

Quality control of the raw data was performed using Fastp software (version 0.19.5) (Chen et al., 2018). Adapter sequences, low-quality bases, sequences containing ambiguous bases, and those shorter than 30 bp were eliminated, resulting in clean reads. These clean reads were aligned to the reference genome (GRCm38.p6) using HiSat2 software (version 2.1.0) (Kim et al., 2019) to determine the overall mapping rate. Subsequently, the number of reads aligned to each gene was calculated using RSEM software (version 1.3.3) (Li and Dewey, 2011). Differential expression analysis between the DSS-treated groups and the control group was conducted using DESeq2 software (version 1.24.0) (Love et al., 2014), employing filtering criteria of |log2FC| ≥ 1 and Padjust <0.05. Pathway enrichment analysis for the set of differentially expressed genes (DEGs) was performed using the Python Scipy software package.

### 2.6 Identifying and validating human homologous genes in UC patients

The names of the interested genes were uploaded to the HomoloGene database (https://www.ncbi.nlm.nih.gov/datasets/gene/) to obtain the corresponding human homologous genes. A search in the GEO database (https://www.ncbi.nlm.nih.gov/geo/) (Barrett et al., 2013) for datasets related to human UC identified GSE224758 as the target dataset. In this dataset, 5 healthy volunteers were designated as the “healthy group,” and 12 UC patients were designated as the “UC group.” The data were further analyzed using GEO2R to determine the regulation patterns of the human homologous genes of interest in UC patients.

### 2.7 Summarizing disease associations of human homologous genes

The names of the interested human homologous genes were queried in the DisGeNET database (https://www.disgenet.com/) (Pinero et al., 2020) to investigate their known associations with various human diseases. Additionally, a literature retrieval was conducted in the Web of Science database to identify recent associations (post-2019) between these genes and human diseases. The findings were meticulously summarized.

### 2.8 Predicting signal peptides and transmembrane helices in human homologous genes

All amino acid sequences corresponding to the human homologous genes were downloaded from the NCBI database. These sequences were then uploaded to the SignalP 6.0 service (https://services.healthtech.dtu.dk/services/SignalP-6.0/) (Almagro et al., 2019; Teufel et al., 2022) for signal peptide prediction and to the DeepTMHMM 1.0 service (https://services.healthtech.dtu.dk/services/DeepTMHMM-1.0/) for transmembrane helices prediction.

### 2.9 Statistical methods

Differences between groups were analyzed using one-way ANOVA followed by Duncan’s multiple comparison test, utilizing SPSS software version 26.0. Spearman’s correlation coefficients were computed, simple regression lines fitted, and visualizations generated using GraphPad Prism version 8.0 software.

## 3 Results

### 3.1 Severity of inflammation in UC model at different DSS concentrations

**FIGURE 1:**
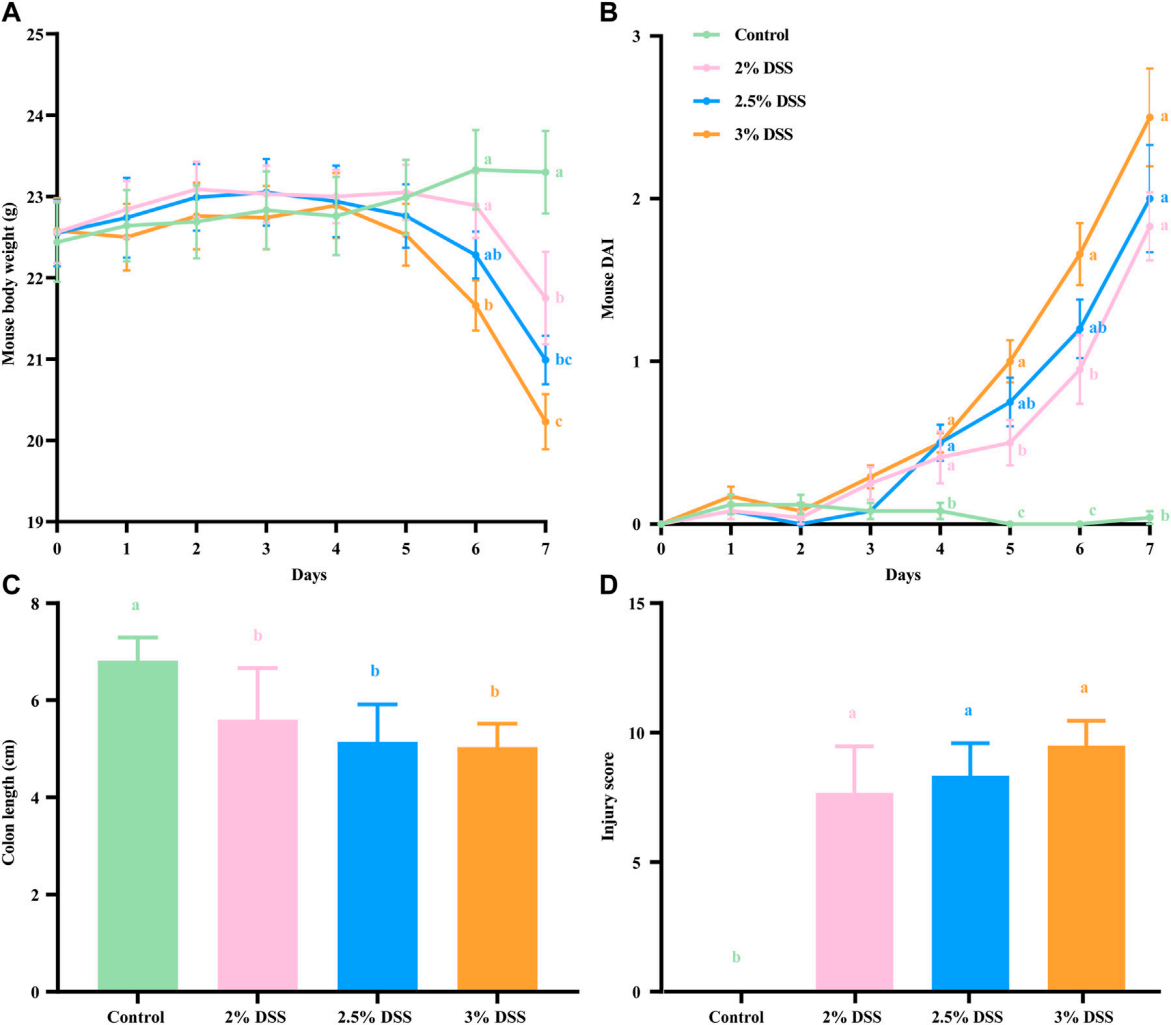
Body weight **(A)**, disease activity index **(B)**, colon length **(C)**, and injury score **(D)** of each group. All results are presented as mean ± standard error (*n* = 8). The legends for Figures A and B are consistent. Groups sharing the same letter do not exhibit significant differences, whereas distinct letters indicate significant variations.

In this study, typical indicators including body weight, disease activity index (DAI), colon length, and injury scores were utilized to evaluate the extent of inflammation in a mouse UC model induced by varying concentrations of DSS. During the experimental period, control group mice exhibited a consistent increase in body weight, whereas a decline was observed on days 4–5 in the three DSS-treated groups. By day 6, the body weight of mice in the 3% DSS group was significantly lower than that of the control group (*p* < 0.01). By day 7, the body weights in all treatment groups were significantly reduced compared to the control (*P* < 0.05 for the 2% DSS group, *P* < 0.01 for both the 2.5% and 3% DSS groups). The loss in final body weight was greatest in the 3% DSS group, followed by the 2.5% and then the 2% DSS group (Figure 1A). Throughout the study, control group mice maintained low DAI values, while those in the treatment groups exhibited a significant increase. From day 4 onwards, DAI values in the treatment groups were significantly higher than those in the control group (*P* < 0.05), with the greatest increase observed in the 3% DSS group, followed by the 2.5% and 2% DSS groups (Figure 1B). Post-experiment dissection revealed significantly shorter colon lengths in the treatment groups compared to the control group (*P* < 0.05 for the 2% DSS group, *P* < 0.01 for the 2.5% DSS group, and *P* < 0.001 for the 3% DSS group), with the magnitude of reduction following the same order (Figure 1C). Injury scores, based on inflammatory cell infiltration, crypt destruction, and lesion extent, were notably higher in the treatment groups than in the control group (*P* < 0.05), again showing an increase correlating with DSS concentration (Figure 1D). These findings collectively demonstrate that the severity of inflammation in the treatment groups escalates with increasing DSS concentration.

### 3.2 Summary of transcriptome sequencing and differentially expressed genes

The raw reads per sample varied between 43,367,718 and 63,292,734, with raw data sizes ranging from 6.55 GB to 9.56 GB and Q30 scores between 93.95% and 94.87%, indicating the high quality of our transcriptome sequencing data. Following quality control, clean reads were obtained, numbering between 43,044,648 and 62,845,704 per sample, resulting in clean data sizes from 6.34 GB to 9.23 GB, with a total of 185.21 GB. Alignment of the clean reads with the reference genome yielded considerable alignment rates ranging from 93.03% to 96.6% per sample (Supplementary Table S1). These mapped reads were predominantly associated with 33,063 known mouse genes.

**FIGURE 2:**
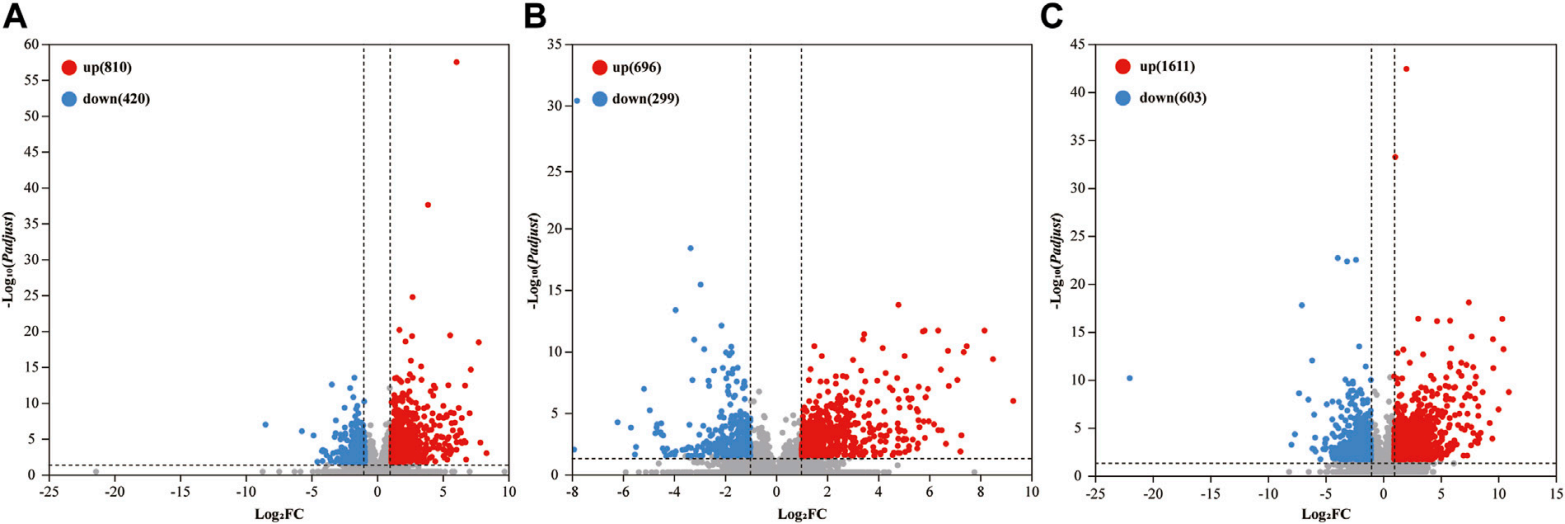
Number of DEGs in the 2% **(A)**, 2.5% **(B)**, and 3% **(C)** DSS-induced groups. The horizontal axis represents log_2_ fold change (log_2_FC), and the vertical axis represents the negative logarithm to the base 10 of the adjusted *p*-value (-log_10_(*Padjust*)). DEGs are identified with criteria of |log_2_FC| ≥ 1 and *Padjust* < 0.05.

**FIGURE 3:**
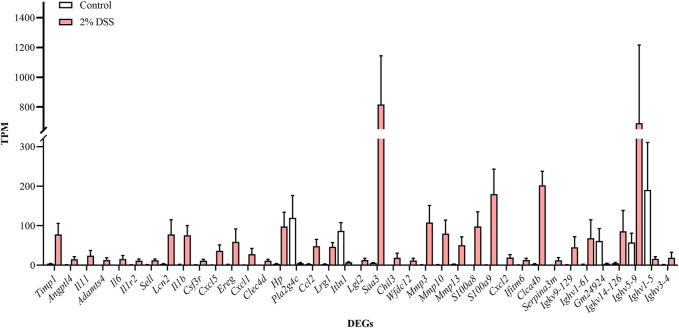
Expression levels of ‘sensitive genes’ for mild UC in the control and 2% DSS-induced mouse group. Data for each gene are expressed as mean ± SEM.

**FIGURE 4:**
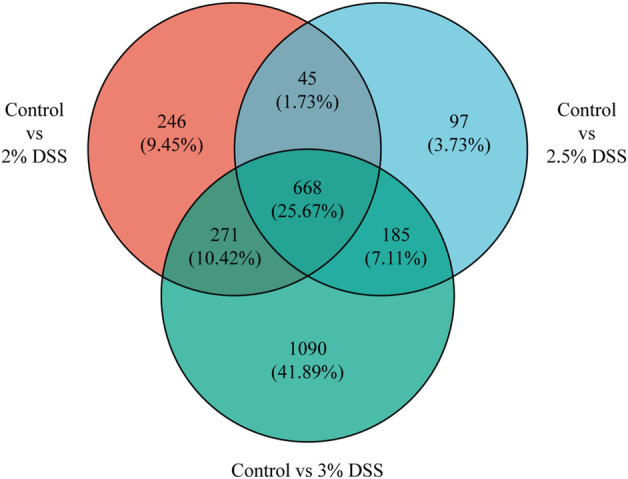
Distribution of shared and unique genes among the three sets of DEGs.

Comparative analysis revealed 810, 696, and 1,611 upregulated differentially expressed genes (DEGs), and 420, 299, and 603 downregulated DEGs in the 2%, 2.5%, and 3% DSS-induced mouse groups, respectively (Figure 2). Notably, compared to the control group, there were 101 DEGs upregulated and 17 DEGs downregulated by more than 10-fold in the 2% DSS-induced mouse group. Among these, 39 DEGs with transcripts per million (TPM) abundance differing by more than 10 between the two groups were defined as “sensitive genes” for mild UC in this study (Figure 3; Supplementary Table S2). Additionally, 668 DEGs were common across all three treatment groups (Figure 4), underscoring their stable regulation under varying DSS concentrations and highlighting their potential role in UC pathogenesis.

### 3.3 KEGG pathway enrichment of shared DEGs and their correlation with DSS concentrations

**FIGURE 5:**
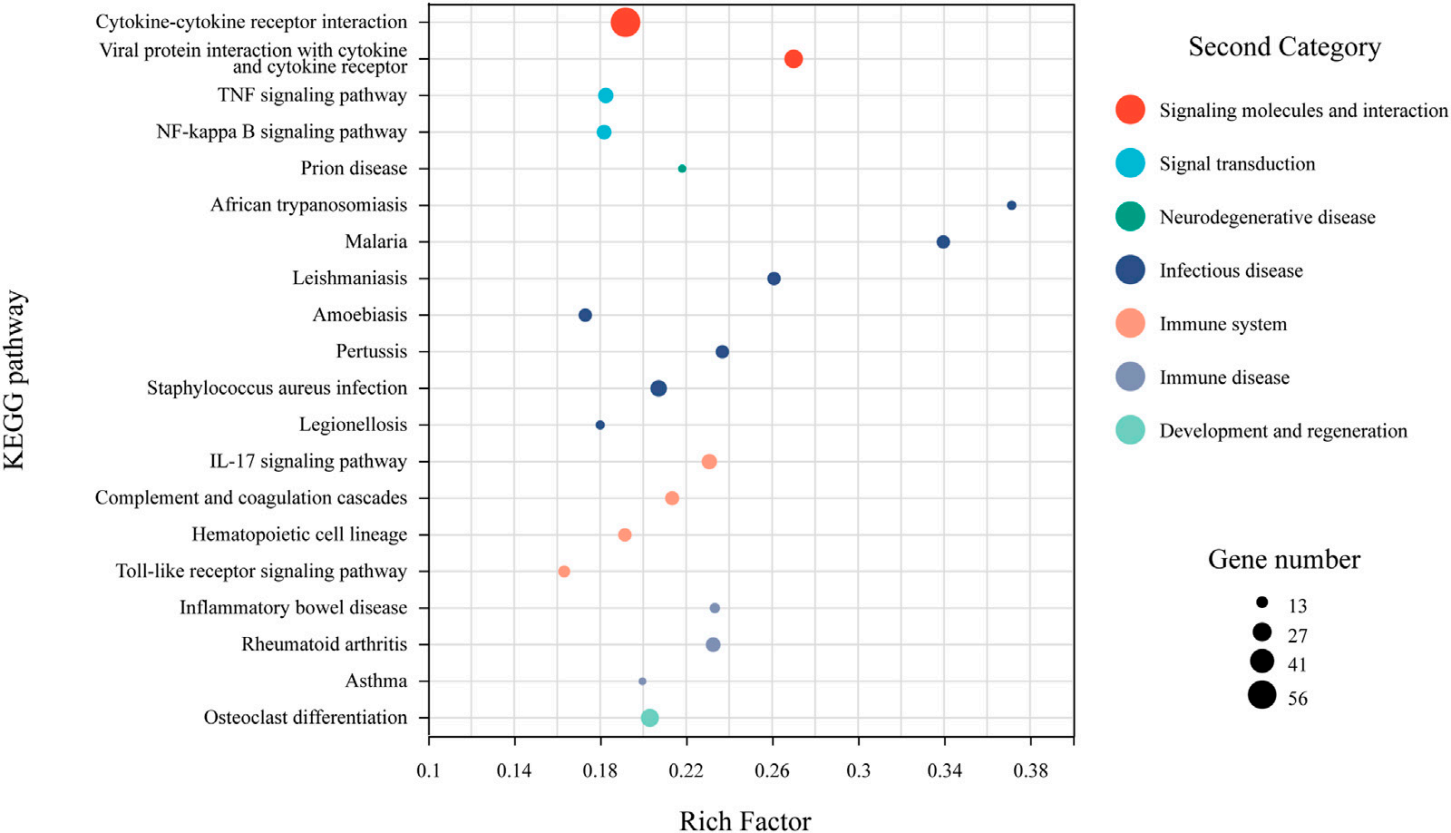
KEGG pathway enrichment analysis for shared DEGs. Dot size reflects the number of genes associated with a given pathway, while dot color indicates the pathway’s classification within the KEGG database. Only the top 20 pathways, based on enrichment factors, are included.

Among the 668 shared DEGs, 440 (65.86%) were annotated using the KEGG database. Pathway enrichment analysis indicated that these genes primarily function in signal transduction, pathogenesis, and immune response (Figure 5). Key pathways within signal transduction included cytokine-cytokine receptor interaction, TNF signaling, and NF-kappa B signaling. For pathogenesis, significant pathways involved malaria and inflammatory bowel disease. In the immune response category, crucial pathways were the IL-17 signaling, Toll-like receptor signaling, and the complement and coagulation cascades.

**FIGURE 6:**
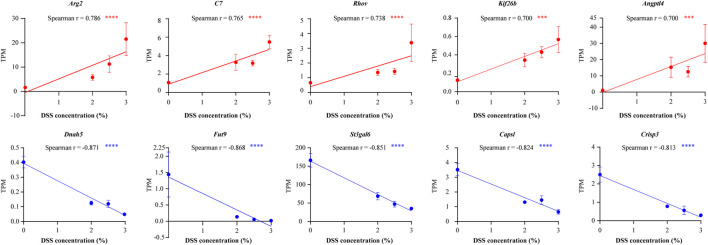
Top five genes showing the strongest positive (red) and strongest negative (blue) correlations with DSS concentrations. Expression levels in each treatment group are presented as mean ± SEM. ^***^ *P* < 0.001, ^****^ *P* < 0.0001.

Spearman correlation analysis revealed that the expression levels of 86% (575/668) of the shared DEGs had a significant correlation with DSS concentration (*P* < 0.05). Notably, 161 of these DEGs had a correlation coefficient with an absolute value greater than 0.6 (Supplementary Table S3), suggesting a strong correlation with UC severity. These were defined as “strong-correlation genes” in this study. The top five positive strong-correlation genes were *Arg2*, *C7*, *Rhov*, *Kif26b*, and *Angptl4*, while the top five negative strong-correlation genes were *Dnah5*, *Fut9*, *St3gal6*, *Capsl*, and *Crisp3* (Figure 6).

### 3.4 Consistently regulated genes in mouse model and UC patients

**FIGURE 7:**
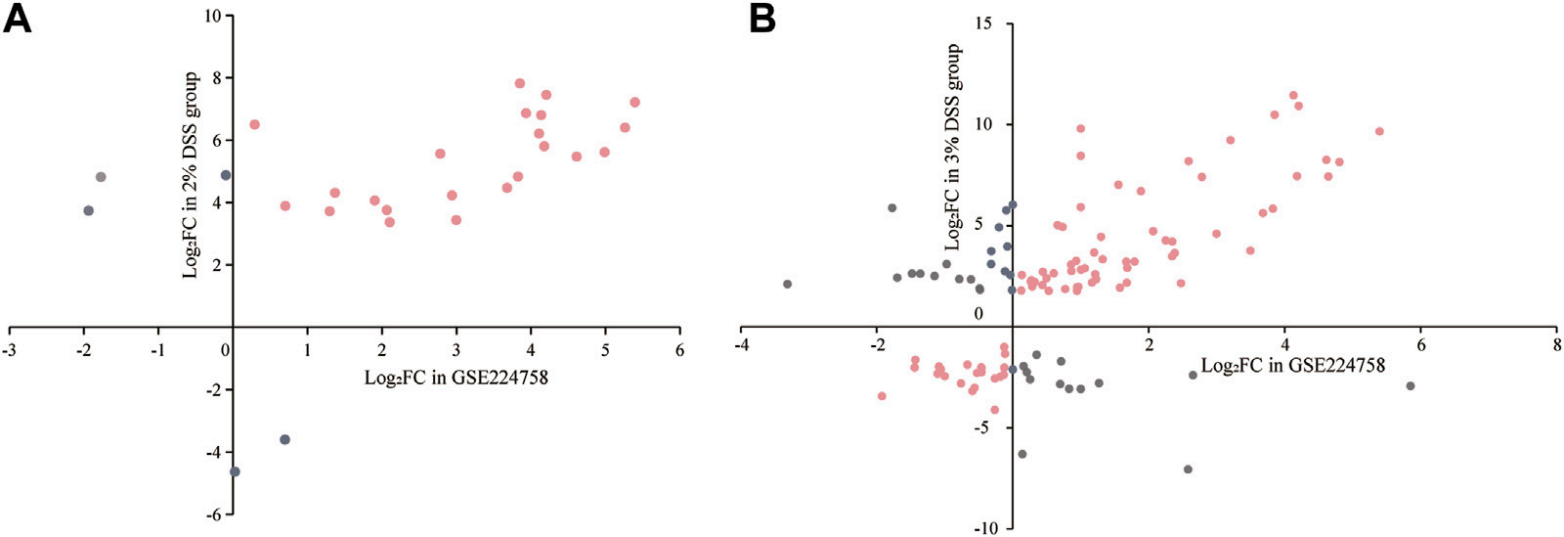
Regulation patterns of “sensitive genes” **(A)** and “strong-correlation genes” **(B)** in the mouse model and UC patients. The horizontal axis represents the log_2_FC values of interested genes in UC patients, and the vertical axis represents their log_2_FC values in the mouse model. Pink points in the first and third quadrants are “consistent genes”; gray points represent “inconsistent genes” and are excluded from further analyses.

Utilizing the GSE224758 dataset, which contains gene expression profile of colon biopsies from 12 UC patients and 5 healthy volunteers, we verified whether the regulation patterns of “sensitive genes” and “strong-correlation genes” in human were consistent with those in the mouse model. There were 27 “sensitive genes” with human homologs. Except for *Hp*, *Il1r2*, *Ereg*, *Itln1*, and *Pla2g4c*, the homologs of the remaining “sensitive genes” (81.5%) showed a consistent regulation pattern in UC patients as observed in the 2% DSS-induced mice group (Figure 7A; Supplementary Table S4). Within the “strong-correlation genes,” 115 had human homologs, of which 81 (70.4%) displayed a consistent regulation pattern in UC patients with those in the 3% DSS-induced mouse group (Figure 7B; Supplementary Table S5). These findings suggest that the DSS-induced mouse model effectively mimics the development pattern of human UC to a large extent. Only these “consistent genes” were retained for further analysis.

### 3.5 Disease associations of “consistent genes”

**FIGURE 8:**
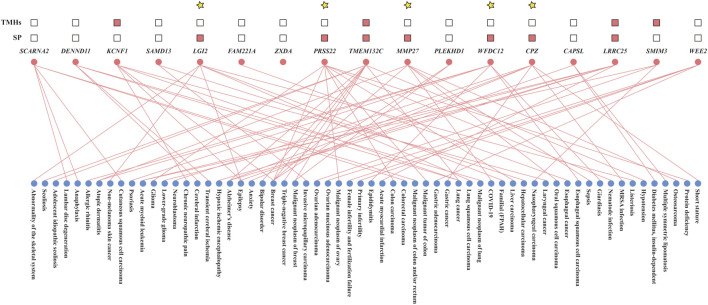
Disease associations and predicted structural features of 17 “more specific genes.” Lines indicate known gene-disease associations; pink squares denote the presence of signal peptides (SP) or transmembrane helices (TMHs). Genes highlighted with yellow asterisks are predicted to produce secretable proteins.

Querying the human homologs of all “consistent genes” in the DisGeNET database revealed that 31 of them, including *Ccl3*, *Slc26a3*, *Col18a1*, *Angptl4*, *Mmp9*, *Cd68*, *Ccl4*, *Rel*, *Pla2g7*, *Ifitm1*, *Lcn2*, *Il1b*, *Il1a*, *Nlrp3*, *Tgm2*, *Trem1*, *S100a8*, *S100a9*, *Cxcl2*, *Clec7a*, *Timp1*, *Il11*, *Il6*, *Sell*, *Cxcl5*, *Cxcl1*, *Ccl2*, *Lrg1*, *Mmp3*, *Mmp10*, and *Mmp13*, had confirmed associations with human UC. The number of human diseases other than UC linked to these confirmed “consistent genes” varied between 41 and 1,824. The remaining 61 “consistent genes,” potentially novel indicators of human UC identified in this study, were associated with 0–438 human diseases (Supplementary Table S6). Combining with the post-2019 literature retrieval, 17 “consistent genes” were found to be associated with fewer than 10 human diseases (Figure 8; Supplementary Table S7), suggesting they might be more specific to human UC than other genes. These were defined as “more specific genes”.

### 3.6 Signal peptides and transmembrane helices in “more specific genes”

For the 17 “consistent genes” that were more specific to human UC identified above, we analyzed the presence of signal peptides and transmembrane helices within the amino acid sequences of their human homologous genes. Ultimately, human genes homologous to *Mmp27*, *Wfdc12*, *Cpz*, *Lgi2*, and *Prss22* were found to habour a signal peptide while lacking transmembrane helices, predicting their translation products as secreted proteins (Figure 8; Supplementary Table S8).

## 4 Discussion

Ulcerative colitis (UC), increasingly recognized as a global health concern, significantly impacts human health and quality of life. This condition results in colonic mucosal damage and histological lesions in the intestine, clinically manifested by symptoms such as weight loss, diarrhea, blood in feces, and reduced colon length. Consequently, indicators such as body weight changes, the Disease Activity Index (DAI), colon length, and injury scores are widely used in colitis research to assess the severity of inflammation in UC (Liu et al., 2021; Deng et al., 2022; Chen et al., 2023; Ren et al., 2023). Based on these indicators, this study assessed inflammation severity in mouse groups treated with 2%, 2.5%, and 3% DSS solutions, revealing a correlation between increased DSS concentrations and heightened inflammation. This finding is consistent with another study employing a gradient of 1.5%, 2.5%, and 3.5% DSS (Almutary et al., 2023), which further confirmed the effectiveness of mouse UC models induced by different DSS concentrations in simulating the progression of UC from mild to severe stages.

As the treatment group with the lowest severity of inflammation in this study, through transcriptome sequencing and comparative analysis, we identified 1230 genes whose expression levels were significantly regulated in the colon tissues of mice belonging to the 2% DSS-induced treatment group in comparison with the control group, predicting their potential as biomarkers for the early diagnosis of UC. In order to select the ones that were more sensitive to early symptoms, we set a higher threshold for fold change as well as a difference in TPM abundance, finally obtaining 39 “sensitive genes” that satisfied the screening conditions. To verify their applicability to human UC, we validated their regulation patterns in the UC patients from dataset GSE224758 and found that 81.5% of the genes were consistent, which were further used as candidates for early diagnostic biomarkers of human UC. Searching DisGeNET, a database of known human gene-disease correlations and evidence, revealed that the human homologs of *Timp1*, *Angptl4*, *Il11*, *Il6*, *Sell*, *Lcn2*, *Il1b*, *Cxcl5*, *Cxcl1*, *Ccl2*, *Lrg1*, *Mmp3*, *Mmp10*, *Mmp13*, *S100a8*, *S100a9*, and *Cxcl2* were associated with human UC (Supplementary Table S6). Among them, *S100a8* and *S100a9* encoding fecal calprotectin (Jukic et al., 2021; Iordache et al., 2023) have been widely used as a biomarkers for human UC. In the DisGeNET database, 298 and 287 human diseases were associated with *S100a8* and *S100a9* (Supplementary Table S6), respectively, which is consistent with the lack of specificity of fecal calprotectin for the diagnosis of UC, as mentioned earlier. These findings suggested that it is highly feasible and credible to search for biomarkers for human UC through the method we performed.

As the DisGeNET database only collected data before 2019, we further investigated relevant reports after 2019 to accurately assess the associations between the above “consistent genes” and human diseases. Ultimately, *LGI2* and *WFDC12* fulfilled the screening criteria. *LGI2* belongs to the leucine-rich glioma-inactivated (LGI) family (Marafi et al., 2022), whose mutations have been associated with human epilepsy (Pakozdy et al., 2015). Further studies have confirmed the overexpression of *LGI2* in patients with lung squamous cell carcinoma (Zeng et al., 2024) and in non-melanoma skin cancer model mice (Sarwar et al., 2023). *WFDC12* has been demonstrated to play an immunomodulatory and anti-inflammatory role through the inhibition of neutrophil elastase (Andrade et al., 2021), and is upregulated in lesional psoriatic skin, wounds, scars (Wang et al., 2019; Zhao et al., 2022), epididymitis in mice (Andrade et al., 2021), and human intestinal epithelial cells under Giardia infections (Rojas et al., 2022). The clinical manifestations of these related diseases, except for Giardia infection which presents as diarrhea, are easily distinguishable from human UC. Moreover, based on the presence of signal peptides and the absence of transmembrane helices, *LGI2* was predicted to be a secreted protein typically detectable in the serum (Vathipadiekal et al., 2015), further satisfying the easy-to-perform characteristic of biomarkers. Altogether, these results suggested that *LGI2* found in this study was a potential biomarker with simultaneous sensitivity, specificity, and ease-to-perform for the early diagnosis of human UC.

There were 668 genes that showed significant differential expression levels in the colon tissues of mice with all three severities of inflammation. Functional enrichment analysis revealed that these DEGs were mainly involved in pathways related to signal transduction, pathogenesis, and immune response processes, including multiple signaling pathways such as TNF, NF-κB, IL-17, and Toll-like receptor, as well as cytokine-cytokine receptor interaction. TNF is a cell signaling protein involved in systemic inflammation (Chu, 2013) and activates NF-κB when cross-linked with its receptor (Seigner et al., 2018). Toll-like receptors also activate NF-κB upon recognition of cellular stress or injury through a cascading signaling process (Patel et al., 2019). NF-κB can modulate the expression levels of several pro-inflammatory cytokines (Akhtar et al., 2020; Gallucci et al., 2022; Karabay et al., 2023), such as IL-17 (He et al., 2022; Wang et al., 2024), which trigger the inflammatory response of the organism through their interaction with the corresponding receptors. Signaling pathways that were significantly modulated during pathogenesis generally received the first consideration as therapeutic targets in biologic therapies. Anti-TNF agents were the first biologic therapies to treat moderate or serve UC (Pugliese et al., 2017). Monoclonal antibodies against various integrins such as IL-12 and IL-23 were another promising biotherapeutic agents (Sands et al., 2019a; Sands et al., 2019b; Sandborn et al., 2020). This suggested that the signaling molecules that were sustainably regulated throughout the whole development process of UC revealed in this study had the potential to serve as biotherapeutic targets.

Given the positive correlation between UC inflammation severity and DSS concentrations, the 161 DEGs whose expression levels were strongly correlated with DSS concentrations were considered to have potential for indicating UC inflammation severity. Of these, only the human homologs of *Mmp27*, *Cpz*, and *Prss22* met both the screening criteria set in this study for disease specificity and ease of detection. Although Spearman correlation analysis indicated a significantly strong positive correlation between their expression levels and the corresponding DSS concentrations, the expression levels of the *Mmp27* and *Cpz* genes in the colon tissues of the 2.5% DSS group mice were lower than those in the 2% DSS group, which may be due to the complex regulatory mechanisms within the organism or the bias of the expression data. The expression level of the remaining *Prss22* gene increased steadily in strict accordance with the DSS concentration gradient, eventually increasing 291-fold compared to the control (Supplementary Table S3). *PRSS22* was initially identified as a member of the serine protease family, which plays essential roles in various physiological processes such as immunity, blood coagulation, cell migration, and the reconstitution of extracellular matrices (Song et al., 2022). During the ongoing development of UC, it will gradually increase the degree of fecal occult blood in the patient. It can be hypothesized that the *PRSS22* gene may act as a coagulation factor in the human organism, and its elevated expression level may be in response to colon bleeding signals. In addition, *PRSS22* was also associated with colorectal cancer (Solmi et al., 2006), revealing its importance in indicating colonic disorders.

By comprehensive consideration of sensitivity, disease specificity, and ease of detection, *LGI2* and *PRSS22* were identified in this study as potential biomarkers for early diagnosis and dynamic severity monitoring in human UC, respectively. To date, these two genes had not been shown to be associated with human UC, suggesting that this study provides new insights into the accurate diagnosis and monitoring of human UC. However, this study still has some limitations, including a relatively small research system, extrapolation based on data analysis only at the mRNA level without further validation, and a range of DSS concentration settings that may not fully encompass both milder and more severe types of UC. In the future, there is a need to validate our inferences in a larger human research system. For example, testing whether serum concentration of LGI2 protein can distinguish patients with mild UC from healthy populations. Determining whether the protein concentration of PRSS22 in the serum of UC patients at different inflammatory severities correlates significantly and strongly with colonoscopic activity, and if so, further delimiting the range of its thresholds at different stages of UC. This will advance their clinical applications in early diagnosis and dynamic monitoring of human UC.

## 5 Conclusion

In conclusion, the present study has identified two potentially novel biomarkers with higher specificity for human UC, which provides new insights into the accurate diagnosis and dynamic monitoring of this persistent disease.

## Data Availability

The original contributions presented in the study are publicly available. This data is deposited in the NCBI repository, accession number SRR27181989-SRR27182012.

## References

[B1] Akhtar M. Guo S. Guo Y. F. Zahoor A. Shaukat A. Chen Y. (2020). Upregulated-gene expression of pro-inflammatory cytokines (TNF-α, IL-1β and IL-6) via TLRs following NF-κB and MAPKs in bovine mastitis. Acta Trop. 207, 105458. 10.1016/j.actatropica.2020.105458 32243879

[B2] Almagro A. J. Tsirigos K. D. Sonderby C. K. Petersen T. N. Winther O. Brunak S. (2019). SignalP 5.0 improves signal peptide predictions using deep neural networks. Nat. Biotechnol. 37, 420–423. 10.1038/s41587-019-0036-z 30778233

[B3] Almutary A. G. Alnuqaydan A. M. Almatroodi S. A. Tambuwala M. M. (2023). Comparative analysis of the effect of different concentrations of dextran sodium sulfate on the severity and extent of inflammation in experimental ulcerative colitis. Appl. Sci. 13, 3233. 10.3390/app13053233

[B4] Alper A. Zhang L. Pashankar D. S. (2017). Correlation of erythrocyte sedimentation rate and C-Reactive protein with pediatric inflammatory bowel disease activity. J. Pediatr. Gastroenterol. Nutr. 65, e25–e27. 10.1097/MPG.0000000000001444 27741061

[B5] Andrade A. D. Almeida P. Mariani N. Freitas G. A. Kushima H. Filadelpho A. L. (2021). Lipopolysaccharide-induced epididymitis modifies the transcriptional profile of Wfdc genes in mice†. Biol. Reprod. 104, 144–158. 10.1093/biolre/ioaa189 33034631

[B6] Arda-Pirincci P. Aykol-Celik G. (2020). Galectin-1 reduces the severity of dextran sulfate sodium (DSS)-induced ulcerative colitis by suppressing inflammatory and oxidative stress response. Bosn. J. Basic Med. Sci. 20, 319–328. 10.17305/bjbms.2019.4539 31999939 PMC7416175

[B7] Barrett T. Wilhite S. E. Ledoux P. Evangelista C. Kim I. F. Tomashevsky M. (2013). NCBI GEO: archive for functional genomics data sets--update. Nucleic Acids Res. 41, D991–D995. 10.1093/nar/gks1193 23193258 PMC3531084

[B8] Burri E. Beglinger C. (2014). The use of fecal calprotectin as a biomarker in gastrointestinal disease. Expert Rev. Gastroenterol. Hepatol. 8, 197–210. 10.1586/17474124.2014.869476 24345070

[B9] Celhar T. Fairhurst A. M. (2017). Modelling clinical systemic lupus erythematosus: similarities, differences and success stories. Rheumatol. Oxf. 56, i88–i99. 10.1093/rheumatology/kew400 PMC5410990 28013204

[B10] Chen L. Zhong X. L. Cao W. Y. Mao M. L. Liu D. D. Liu W. J. (2023). IGF2/IGF2R/Sting signaling as a therapeutic target in DSS-induced ulcerative colitis. Eur. J. Pharmacol. 960, 176122. 10.1016/j.ejphar.2023.176122 37863414

[B11] Chen S. Zhou Y. Chen Y. Gu J. (2018). Fastp: an ultra-fast all-in-one FASTQ preprocessor. Bioinformatics 34, i884–i890. 10.1093/bioinformatics/bty560 30423086 PMC6129281

[B12] Chu W. M. (2013). Tumor necrosis factor. Cancer Lett. 328, 222–225. 10.1016/j.canlet.2012.10.014 23085193 PMC3732748

[B13] Costa M. J. Kudaravalli J. Liu W. H. Stock J. Kong S. Liu S. H. (2018). A mouse model for evaluation of efficacy and concomitant toxicity of anti-human CXCR4 therapeutics. PLoS One 13, e0194688. 10.1371/journal.pone.0194688 29554149 PMC5858835

[B14] Deng L. Guo H. Wang S. Liu X. Lin Y. Zhang R. (2022). The attenuation of chronic ulcerative colitis by (R)-salbutamol in repeated DSS-Induced mice. Oxidative Med. Cell. Longev. 2022, 9318721–9318820. 10.1155/2022/9318721 PMC8843997 35178163

[B15] de Voogd F. van Wassenaer E. A. Mookhoek A. Bots S. van Gennep S. Löwenberg M. (2022). Intestinal ultrasound is accurate to determine endoscopic response and remission in patients with moderate to severe ulcerative colitis: a longitudinal prospective cohort study. Gastroenterology 163, 1569–1581. 10.1053/j.gastro.2022.08.038 36030056

[B16] Gallucci G. M. Alsuwayt B. Auclair A. M. Boyer J. L. Assis D. N. Ghonem N. S. (2022). Fenofibrate downregulates NF-κB signaling to inhibit pro-inflammatory cytokine secretion in human THP-1 macrophages and during primary biliary cholangitis. Inflammation 45, 2570–2581. 10.1007/s10753-022-01713-1 35838934 PMC10853883

[B17] Golovics P. A. Gonczi L. Reinglas J. Verdon C. Pundir S. Afif W. (2022). Patient-Reported outcome and clinical scores are equally accurate in predicting mucosal healing in ulcerative colitis: a prospective study. Dig. Dis. Sci. 67, 3089–3095. 10.1007/s10620-021-07178-w 34286411

[B18] Gros B. Kaplan G. G. (2023). Ulcerative colitis in adults: a review. Jama 330, 951–965. 10.1001/jama.2023.15389 37698559

[B19] Guo M. Wang X. (2023). Pathological mechanism and targeted drugs of ulcerative colitis: a review. Med. Baltim. 102, e35020. 10.1097/MD.0000000000035020 PMC10508406 37713856

[B20] He X. Song X. Cao H. Zhou Q. Zhang J. Yue H. (2022). Glaesserella parasuis induces IL-17 production might through PKC-ERK/MAPK and IκB/NF-κB signaling pathways. Vet. Microbiol. 273, 109521. 10.1016/j.vetmic.2022.109521 35932516

[B21] Huang S. Fu Y. Xu B. Liu C. Wang Q. Luo S. (2020). Wogonoside alleviates colitis by improving intestinal epithelial barrier function via the MLCK/pMLC2 pathway. Phytomedicine 68, 153179. 10.1016/j.phymed.2020.153179 32062328

[B22] Iordache M. M. Belu A. M. Vlad S. E. Aivaz K. A. Dumitru A. Tocia C. (2023). Calprotectin, biomarker of depression in patients with inflammatory bowel disease? Med. Kaunas. 59, 1240. 10.3390/medicina59071240 PMC10383955 37512053

[B23] Ishida N. Higuchi T. Miyazu T. Tamura S. Tani S. Yamade M. (2021). C-reactive protein is superior to fecal biomarkers for evaluating colon-wide active inflammation in ulcerative colitis. Sci. Rep. 11, 12431. 10.1038/s41598-021-90558-z 34127687 PMC8203605

[B24] Jukic A. Bakiri L. Wagner E. F. Tilg H. Adolph T. E. (2021). Calprotectin: from biomarker to biological function. Gut 70, 1978–1988. 10.1136/gutjnl-2021-324855 34145045 PMC8458070

[B25] Jung J. H. Ko J. Lee E. H. Choi K. M. Kim M. Yim U. H. (2017). RNA seq- and DEG-based comparison of developmental toxicity in fish embryos of two species exposed to Iranian heavy crude oil. Comp. Biochem. Physiol. C Toxicol. Pharmacol. 196, 1–10. 10.1016/j.cbpc.2017.02.010 28257923

[B26] Justice M. J. Dhillon P. (2016). Using the mouse to model human disease: increasing validity and reproducibility. Dis. Model. Mech. 9, 101–103. 10.1242/dmm.024547 26839397 PMC4770152

[B27] Kaplan G. G. Ng S. C. (2017). Understanding and preventing the global increase of inflammatory bowel disease. Gastroenterology 152, 313–321.e2. 10.1053/j.gastro.2016.10.020 27793607

[B28] Karabay O. Guney E. G. Alkurt U. Hamarat K. F. Deveci O. A. Aydin A. (2023). The predictive role of NF-κB-mediated pro-inflammatory cytokine expression levels in hepatitis B vaccine response. J. Immunoass. Immunochem. 44, 192–203. 10.1080/15321819.2022.2164507 36656054

[B29] Kawashima K. Oshima N. Kishimoto K. Kataoka M. Fukunaga M. Kotani S. (2023). Low fecal calprotectin predicts histological healing in patients with ulcerative colitis with endoscopic remission and leads to prolonged clinical remission. Inflamm. Bowel Dis. 29, 359–366. 10.1093/ibd/izac095 35583193

[B30] Kim D. Paggi J. M. Park C. Bennett C. Salzberg S. L. (2019). Graph-based genome alignment and genotyping with HISAT2 and HISAT-genotype. Nat. Biotechnol. 37, 907–915. 10.1038/s41587-019-0201-4 31375807 PMC7605509

[B31] Kohli M. Young C. Y. Tindall D. J. Nandy D. McKenzie K. M. Bevan G. H. (2015). Whole blood defensin mRNA expression is a predictive biomarker of docetaxel response in castration-resistant prostate cancer. Onco Targets Ther. 8, 1915–1922. 10.2147/OTT.S86637 26261420 PMC4527520

[B32] Kuenzig M. E. Manuel D. G. Donelle J. Benchimol E. I. (2020). Life expectancy and health-adjusted life expectancy in people with inflammatory bowel disease. Cmaj 192, E1394-E1402–E1402. 10.1503/cmaj.190976 33168761 PMC7669301

[B33] Le Berre C. Honap S. Peyrin-Biroulet L. (2023). Ulcerative colitis. Lancet 402, 571–584. 10.1016/S0140-6736(23)00966-2 37573077

[B34] Li B. Dewey C. N. (2011). RSEM: accurate transcript quantification from RNA-Seq data with or without a reference genome. BMC Bioinforma. 12, 323. 10.1186/1471-2105-12-323 PMC3163565 21816040

[B35] Li C. J. Wang Y. K. Zhang S. M. Ren M. D. He S. X. (2023). Global burden of inflammatory bowel disease 1990-2019: a systematic examination of the disease burden and twenty-year forecast. World J. Gastroenterol. 29, 5751–5767. 10.3748/wjg.v29.i42.5751 38075848 PMC10701338

[B36] Li H. Shen L. Lv T. Wang R. Zhang N. Peng H. (2019). Salidroside attenuates dextran sulfate sodium-induced colitis in mice via SIRT1/FoxOs signaling pathway. Eur. J. Pharmacol. 861, 172591. 10.1016/j.ejphar.2019.172591 31401159

[B37] Liang J. Lv J. Liu Z. (2015). Identification of stage-specific biomarkers in lung adenocarcinoma based on RNA-seq data. Tumour Biol. 36, 6391–6399. 10.1007/s13277-015-3327-0 25861020

[B38] Lin G. Li M. Xu N. Wu X. Liu J. Wu Y. (2020). Anti-inflammatory effects of *Heritiera littoralis* fruits on dextran sulfate sodium- (DSS-) induced ulcerative colitis in mice by regulating gut microbiota and suppressing NF-*κ*B pathway. Biomed. Res. Int. 2020, 8893621. 10.1155/2020/8893621 33354574 PMC7735845

[B39] Liu Y. Zhou M. Yang M. Jin C. Song Y. Chen J. (2021). Pulsatilla chinensis saponins ameliorate inflammation and DSS-Induced ulcerative colitis in rats by regulating the composition and diversity of intestinal flora. Front. Cell. Infect. Microbiol. 11, 728929. 10.3389/fcimb.2021.728929 34804990 PMC8602866

[B40] Love M. I. Huber W. Anders S. (2014). Moderated estimation of fold change and dispersion for RNA-seq data with DESeq2. Genome Biol. 15, 550. 10.1186/s13059-014-0550-8 25516281 PMC4302049

[B41] Marafi D. Kozar N. Duan R. Bradley S. Yokochi K. Al Mutairi F. (2022). A reverse genetics and genomics approach to gene paralog function and disease: myokymia and the juxtaparanode. Am. J. Hum. Genet. 109, 1713–1723. 10.1016/j.ajhg.2022.07.006 35948005 PMC9502070

[B42] Mutryn M. F. Brannick E. M. Fu W. Lee W. R. Abasht B. (2015). Characterization of a novel chicken muscle disorder through differential gene expression and pathway analysis using RNA-sequencing. BMC Genomics 16, 399. 10.1186/s12864-015-1623-0 25994290 PMC4438523

[B43] Ng W. K. Wong S. H. Ng S. C. (2016). Changing epidemiological trends of inflammatory bowel disease in Asia. Intest. Res. 14, 111–119. 10.5217/ir.2016.14.2.111 27175111 PMC4863044

[B44] Pakozdy A. Patzl M. Zimmermann L. Jokinen T. S. Glantschnigg U. Kelemen A. (2015). LGI proteins and epilepsy in human and animals. J. Vet. Intern Med. 29, 997–1005. 10.1111/jvim.12610 26032921 PMC4895363

[B45] Patel B. Banerjee R. Basu M. Lenka S. S. Paichha M. Samanta M. (2019). Toll like receptor induces Ig synthesis in Catla catla by activating MAPK and NF-κB signalling. Mol. Immunol. 105, 62–75. 10.1016/j.molimm.2018.11.012 30496978

[B46] Pinero J. Ramirez-Anguita J. M. Sauch-Pitarch J. Ronzano F. Centeno E. Sanz F. (2020). The DisGeNET knowledge platform for disease genomics: 2019 update. Nucleic Acids Res. 48, D845-D855–D855. 10.1093/nar/gkz1021 31680165 PMC7145631

[B47] Pugliese D. Felice C. Papa A. Gasbarrini A. Rapaccini G. L. Guidi L. (2017). Anti TNF-α therapy for ulcerative colitis: current status and prospects for the future. Expert Rev. Clin. Immunol. 13, 223–233. 10.1080/1744666X.2017.1243468 27687496

[B48] Ren R. Zhao A. Q. Chen L. Wu S. Hung W. L. Wang B. (2023). Therapeutic effect of Lactobacillus plantarum JS19 on mice with dextran sulfate sodium induced acute and chronic ulcerative colitis. J. Sci. Food Agric. 103, 4143–4156. 10.1002/jsfa.12414 36573836

[B49] Rojas L. Gruttner J. Ma'Ayeh S. Xu F. Svard S. G. (2022). Dual RNA sequencing reveals key events when different giardia life cycle stages interact with human intestinal epithelial cells *in vitro* . Front. Cell. Infect. Microbiol. 12, 862211. 10.3389/fcimb.2022.862211 35573800 PMC9094438

[B50] Sandborn W. J. Baert F. Danese S. Krznaric Z. Kobayashi T. Yao X. (2020). Efficacy and safety of vedolizumab subcutaneous formulation in a randomized trial of patients with ulcerative colitis. Gastroenterology 158, 562–572. 10.1053/j.gastro.2019.08.027 31470005

[B51] Sands B. E. Peyrin-Biroulet L. Loftus E. J. Danese S. Colombel J. F. Törüner M. (2019a). Vedolizumab versus adalimumab for moderate-to-severe ulcerative colitis. N. Engl. J. Med. 381, 1215–1226. 10.1056/NEJMoa1905725 31553834

[B52] Sands B. E. Sandborn W. J. Panaccione R. O'Brien C. D. Zhang H. Johanns J. (2019b). Ustekinumab as induction and maintenance therapy for ulcerative colitis. N. Engl. J. Med. 381, 1201–1214. 10.1056/NEJMoa1900750 31553833

[B53] Sarkar S. Heise M. T. (2019). Mouse models as resources for studying infectious diseases. Clin. Ther. 41, 1912–1922. 10.1016/j.clinthera.2019.08.010 31540729 PMC7112552

[B54] Sarwar M. S. Ramirez C. N. Dina K. H. Chou P. Wu R. Sargsyan D. (2023). The environmental carcinogen benzo[a]pyrene regulates epigenetic reprogramming and metabolic rewiring in a two-stage mouse skin carcinogenesis model. Carcinogenesis 44, 436–449. 10.1093/carcin/bgad024 37100755 PMC10414144

[B55] Satoh J. Yamamoto Y. Asahina N. Kitano S. Kino Y. (2014). RNA-Seq data mining: downregulation of NeuroD6 serves as a possible biomarker for alzheimer's disease brains. Dis. Markers 2014, 123165. 10.1155/2014/123165 25548427 PMC4274867

[B56] Seigner J. Basilio J. Resch U. de Martin R. (2018). CD40L and TNF both activate the classical NF-κB pathway, which is not required for the CD40L induced alternative pathway in endothelial cells. Biochem. Biophys. Res. Commun. 495, 1389–1394. 10.1016/j.bbrc.2017.11.160 29183724

[B57] Singh S. Ananthakrishnan A. N. Nguyen N. H. Cohen B. L. Velayos F. S. Weiss J. M. (2023). AGA clinical practice guideline on the role of biomarkers for the management of ulcerative colitis. Gastroenterology 164, 344–372. 10.1053/j.gastro.2022.12.007 36822736

[B58] Solmi R. Ugolini G. Rosati G. Zanotti S. Lauriola M. Montroni I. (2006). Microarray-based identification and RT-PCR test screening for epithelial-specific mRNAs in peripheral blood of patients with colon cancer. BMC Cancer 6, 250. 10.1186/1471-2407-6-250 17054783 PMC1629022

[B59] Song L. Li H. Ma R. R. Liu S. Zhang G. H. Guo X. Y. (2022). E2F1-initiated transcription of PRSS22 promotes breast cancer metastasis by cleaving ANXA1 and activating FPR2/ERK signaling pathway. Cell. Death Dis. 13, 982. 10.1038/s41419-022-05414-3 36414640 PMC9681780

[B60] Spiceland C. M. Lodhia N. (2018). Endoscopy in inflammatory bowel disease: role in diagnosis, management, and treatment. World J. Gastroenterol. 24, 4014–4020. 10.3748/wjg.v24.i35.4014 30254405 PMC6148432

[B61] Studd C. Cameron G. Beswick L. Knight R. Hair C. McNeil J. (2016). Never underestimate inflammatory bowel disease: high prevalence rates and confirmation of high incidence rates in Australia. J. Gastroenterol. Hepatol. 31, 81–86. 10.1111/jgh.13050 26222770

[B62] Teufel F. Almagro A. J. Johansen A. R. Gislason M. H. Pihl S. I. Tsirigos K. D. (2022). SignalP 6.0 predicts all five types of signal peptides using protein language models. Nat. Biotechnol. 40, 1023–1025. 10.1038/s41587-021-01156-3 34980915 PMC9287161

[B63] Turner D. Ricciuto A. Lewis A. D'Amico F. Dhaliwal J. Griffiths A. M. (2021). STRIDE-II: an update on the selecting therapeutic targets in inflammatory bowel disease (STRIDE) initiative of the international organization for the study of IBD (IOIBD): determining therapeutic goals for Treat-to-Target strategies in IBD. Gastroenterology 160, 1570–1583. 10.1053/j.gastro.2020.12.031 33359090

[B64] Vathipadiekal V. Wang V. Wei W. Waldron L. Drapkin R. Gillette M. (2015). Creation of a human secretome: a novel composite library of human secreted proteins: validation using ovarian cancer gene expression data and a virtual secretome array. Clin. Cancer Res. 21, 4960–4969. 10.1158/1078-0432.CCR-14-3173 25944803

[B65] Wang H. Pan F. Liu J. Zhang J. Fuli Z. Wang Y. (2024). Huayuwendan decoction ameliorates inflammation via IL-17/NF-κB signaling pathway in diabetic rats. J. Ethnopharmacol. 319, 117328. 10.1016/j.jep.2023.117328 37865275

[B66] Wang Z. Zheng H. Zhou H. Huang N. Wei X. Liu X. (2019). Systematic screening and identification of novel psoriasis-specific genes from the transcriptome of psoriasis-like keratinocytes. Mol. Med. Rep. 19, 1529–1542. 10.3892/mmr.2018.9782 30592269 PMC6390042

[B67] Yang J. W. Zheng D. J. Cui B. D. Yang M. Chen Y. Z. (2016). RNA-seq transcriptome analysis of a Pseudomonas strain with diversified catalytic properties growth under different culture medium. Microbiologyopen 5, 626–636. 10.1002/mbo3.357 27061463 PMC4985596

[B68] Ye X. Huai J. Ding J. (2018). Diagnostic accuracy of fecal calprotectin for screening patients with colorectal cancer: a meta-analysis. Turk J. Gastroenterol. 29, 397–405. 10.5152/tjg.2018.17606 30249553 PMC6284629

[B69] Yoon J. Y. Park S. J. Hong S. P. Kim T. I. Kim W. H. Cheon J. H. (2014). Correlations of C-reactive protein levels and erythrocyte sedimentation rates with endoscopic activity indices in patients with ulcerative colitis. Dig. Dis. Sci. 59, 829–837. 10.1007/s10620-013-2907-3 24352705

[B70] Yu Y. R. Rodriguez J. R. (2017). Clinical presentation of Crohn's, ulcerative colitis, and indeterminate colitis: symptoms, extraintestinal manifestations, and disease phenotypes. Semin. Pediatr. Surg. 26, 349–355. 10.1053/j.sempedsurg.2017.10.003 29126502

[B71] Zeng L. Li L. Liao X. Zhang L. Yin C. Chen X. (2024). Population-based high-dimensional analyses identify multiple intrinsic characters for cancer vaccines against lung squamous cell carcinoma. Med. Oncol. 41, 42. 10.1007/s12032-023-02214-3 38170412

[B72] Zhao F. Zhang C. Li G. Zheng H. Gu L. Zhou H. (2022). A role for whey acidic protein four-disulfide-core 12 (WFDC12) in the pathogenesis and development of psoriasis disease. Front. Immunol. 13, 873720. 10.3389/fimmu.2022.873720 36148224 PMC9485559

[B73] Zhou J. L. Bao J. C. Liao X. Y. Chen Y. J. Wang L. W. Fan Y. Y. (2023). Trends and projections of inflammatory bowel disease at the global, regional and national levels, 1990-2050: a bayesian age-period-cohort modeling study. BMC Public Health 23, 2507. 10.1186/s12889-023-17431-8 38097968 PMC10722679

